# Machine Learning for the Identification of Key Predictors to Bayley Outcomes: A Preterm Cohort Study

**DOI:** 10.3390/jpm14090922

**Published:** 2024-08-30

**Authors:** Petra Grđan Stevanović, Nina Barišić, Iva Šunić, Ann-Marie Malby Schoos, Branka Bunoza, Ruža Grizelj, Ana Bogdanić, Ivan Jovanović, Mario Lovrić

**Affiliations:** 1 Department of Pediatrics, University Hospital Centre Zagreb, Kišpatićeva 12, 10000 Zagreb, Croatia; 2 School of Medicine, University of Zagreb, 10000 Zagreb, Croatia; 3 Centre for Bioanthropology, Institute for Anthropological Research, 10000 Zagreb, Croatia; 4 Copenhagen Prospective Studies on Asthma in Childhood, Herlev and Gentofte Hospital, University of Copenhagen, 2200 Copenhagen, Denmark; 5 Department of Pediatrics, Slagelse Hospital, 4200 Slagelse, Denmark; 6 Faculty of Health and Medical Sciences, Department of Clinical Medicine, University of Copenhagen, 2200 Copenhagen, Denmark; 7 Department of Neuroradiology, University Hospital Centre Zagreb, 10000 Zagreb, Croatia; 8 The Lisbon Council, IPC-Résidence Palace, 1040 Brussels, Belgium

**Keywords:** preterm infants, machine learning, Bayley score, neurodevelopment, sepsis

## Abstract

Background: The aim of this study was to understand how neurological development of preterm infants can be predicted at earlier stages and explore the possibility of applying personalized approaches. Methods: Our study included a cohort of 64 preterm infants, between 24 and 34 weeks of gestation. Linear and nonlinear models were used to evaluate feature predictability to Bayley outcomes at the corrected age of 2 years. The outcomes were classified into motor, language, cognitive, and socio-emotional categories. Pediatricians’ opinions about the predictability of the same features were compared with machine learning. Results: According to our linear analysis sepsis, brain MRI findings and Apgar score at 5th minute were predictive for cognitive, Amiel-Tison neurological assessment at 12 months of corrected age for motor, while sepsis was predictive for socio-emotional outcome. None of the features were predictive for language outcome. Based on the machine learning analysis, sepsis was the key predictor for cognitive and motor outcome. For language outcome, gestational age, duration of hospitalization, and Apgar score at 5th minute were predictive, while for socio-emotional, gestational age, sepsis, and duration of hospitalization were predictive. Pediatricians’ opinions were that cardiopulmonary resuscitation is the key predictor for cognitive, motor, and socio-emotional, but gestational age for language outcome. Conclusions: The application of machine learning in predicting neurodevelopmental outcomes of preterm infants represents a significant advancement in neonatal care. The integration of machine learning models with clinical workflows requires ongoing education and collaboration between data scientists and healthcare professionals to ensure the models’ practical applicability and interpretability.

## 1. Introduction

The continuing advancement of neonatal care correlates with reduced preterm mortality rates and a higher likelihood of survival into adulthood [1]. Infants born before 37 weeks of gestation have an increased susceptibility to adverse neurodevelopmental outcomes related to motor, language, sensory, intellectual, or behavioral development with 70–80 times greater risk for cerebral palsy, compared to term-born infants [2,3,4]. The literature shows that around 17% of preterm infants develop intellectual impairment and 8% have a deficit in language skills [5]. Some of the predictors for adverse neurodevelopmental outcomes in preterm infants are earlier gestational age (GA), lower birth weight (BW), bronchopulmonary dysplasia (BPD), respiratory distress syndrome (RDS), retinopathy of prematurity (ROP), use of caffeine citrate, lower socioeconomic status, level of mother’s education, length of stay in the hospital, higher SNAP II (Score for Neonatal Acute Physiology) or SNAPPE II score (SNAP Perinatal Extension), and discontinuity on electroencephalogram (EEG) recorded on term equivalent age [6]. White matter injuries (WMI) caused by ischemia and infection/inflammation occur more often in preterm infants. Magnetic resonance imaging (MRI) is considered the gold standard in preterm infant neuroimaging. Compared to brain ultrasound, it has an increased sensitivity in detecting non-cystic WMI [7]. Lesions of periventricular crossroads of projection, commissural, and associative fibers located along the lateral wall of ventricles are associated with impaired motor, sensory, and cognitive development [8]. Not visible/slightly visible frontal and parietal crossroads of pathways on term equivalent MRI are associated with unfavorable neurodevelopmental outcomes [8,9]. Abnormal white matter maturation with ventriculomegaly is correlated with poorer motor and language development at the age of two years [10].

Machine learning (ML) models are algorithms that, if trained well, can predict outcomes on unseen patients. Predicting the neurodevelopmental outcome of a preterm infant is a substantial concern for clinicians and caregivers. Early and accurate identification of high-risk preterm infants enables effective treatment interventions, personalized rehabilitation approaches, and educational support to mitigate neurocognitive disabilities as much as possible.

In this work, we investigate the predictive capacity of clinical features, information on brain structure (brain MRI), function (conventional EEG, cEEG), and neurological assessment in relation to neurodevelopmental outcome, as measured by the Bayley Scales of Infant and Toddler Development III (Bayley-III) across all four domains: motor, cognitive, language, and socio-emotional. The goal was to assess which of these variables, individually or combined, are predictive for Bayley-III scores at the age of 2 years. The approach was based on ML and statistical analysis, facilitating the identification of features discerned during early stages that harbor predictive potential with respect to developmental outcomes. The predictability of features obtained by the ML model was compared with general pediatric opinion.

## 2. Materials and Methods

### 2.1. Study Participants

We randomly selected 72 preterm infants who were admitted for treatment at the Neonatal Intensive Care Unit (NICU), University Hospital Centre Zagreb, between September 2014 and July 2019. All premature infants were outborn. Exclusion criteria were the presence of congenital malformations, and chromosomal/other genetic or metabolic disorders that might influence neurological outcomes. Additionally, preterm infants who did not undergo brain MRI at the exact term-equivalent age (40 ± 2 weeks) were excluded from the study. Finally, 64 preterm infants, between 24 + 3 and 34 + 0 weeks of gestation, were included in the study. Written informed consent was obtained from the parents of all participating infants. The Institutional Review Board of the University of Zagreb School of Medicine approved the study in accordance with the Declaration of Helsinki.

### 2.2. Procedures and Methods

We collected comprehensive information about clinical characteristics, brain structure (MRI), and function (cEEG) data, and neurological assessments of preterm infants. The study included preterm infants with all degrees of BPD (according to Jobe and Bancalari classification), NEC or ROP, as well as preterm infants who required minimal respiratory assistance at birth or cardiopulmonary resuscitation. Neuromotor assessments were performed at intervals of 3 months until the corrected age (CA) of two years. The standardized Amiel-Tison examination was performed by an experienced pediatric neurologist (NB, BB) and a pediatric neurology training resident (PGS). Children without neurological difficulties/disturbance or with mild motor/reflex abnormalities were classified as favorable outcomes. Conversely, children demonstrating substantial motor and functional impairments, developmental delays, or signs of central nervous system depression were classified as unfavorable outcomes [11,12,13].

The definition of epileptic seizures includes clinical features relevant to this age group (motor, non-motor, or sequential presentation) and EEG changes according to the ILAE classification and modification for seizures in neonates (2021), emphasizing the role of EEG in the diagnosis [14]. A conventional, 10–20 international EEG bipolar longitudinal placement montage, modified for neonates was used. EEG findings were classified as normal or abnormal and were analyzed by experienced pediatric neurologists (NB, BB) and a pediatric neurology training resident (PGS). In all children, brain MRI was performed at term-equivalent age (40 ± 2) weeks, primarily using the ”feed and wrap” technique [15]. In some children, additional sedation with phenobarbitone was required. A 3T MRI scanner was used as a standard neuroimaging procedure. MR scans were analyzed by a neuroradiologist (IJ) blinded to the medical data. We evaluated the visibility of frontal and parietal crossroads based on histological and MRI descriptions of transient fetal structures. Visible or positively recognized crossroads were presented as high signal intensity on T2-weighted MRI scans [8,16,17]. Dilatation of the lateral ventricle (LV) was assessed by measuring the LV diameter on the coronal section at the level of the ventricular atrium [18]. Ventriculomegaly was diagnosed if the LV diameter was >10 mm. Focal white matter injuries were presented with T1 hyperintensity in the absence of T2 hypointensity [19]. Finally, the total MR finding was classified as normal or abnormal (presence of ventriculomegaly, focal white matter injury, or poor/no visibility of periventricular crossroads).

Assessments with the Bayley-III scale were conducted when the infants reached the age of 2 years of CA by the same experienced psychologist (AB) in a calm environment. To avoid interference with the Bayley evaluation, the collection of clinical and demographic data was performed after testing [20,21,22,23,24].

### 2.3. Data Preparation

In total, 30 predictive variables were included and listed in Table 1. The outcome variables were Bayley III categories (cognitive, language, motor, and socio-emotional). Analyses were performed using the Bayley scores as either a continuous value (45–140) or a dichotomized value (cutoff 85). Those with 85 and higher were assigned as number 0 (normal) and those with lower values as number 1 (abnormal).

### 2.4. Statistical Analysis and Machine Learning

Linear and nonlinear models were used alongside univariate statistical tests. All data analyses were performed with the programming language Python (v.3.8.16.). For the analyses, the following libraries were utilized as well: SciPy [25], statsmodels [26] and Pingouin [27] libraries, while graphs were created with Matplotlib and Seaborn libraries [28,29]. Univariate statistical tests were applied to assess the differences in Bayley-III scores and provide further insight into the predictive quality of the clinical variables. Depending on the type of outcome, we applied different statistical methods for the univariate tests. For the original continuous Bayley-III scores, we used Spearman correlation when comparing them with continuous independent predictors. To analyze the dichotomized Bayley-III scores (0, 1 scale with a cutoff at 85) with independent predictors that followed a normal distribution, we applied the *t*-test. For other predictors, associations with the dichotomized Bayley-III scores were analyzed using Fisher’s exact test. We focused on variables with *p*-values lower than 0.01 (*p* < 0.01) to determine statistical significance.

For the multivariate analysis, linear regression was used to examine the continuous Bayley scores. In contrast, logistic regression, and machine learning by means of Random Forests (RF) were used to investigate the associations with the dichotomized Bayley scores. A significant level of 0.05 was used for logistic and linear regression. The data set was decorrelated (above 85% correlation) and variables with rare binary values were removed from the data.

**Figure 1:**
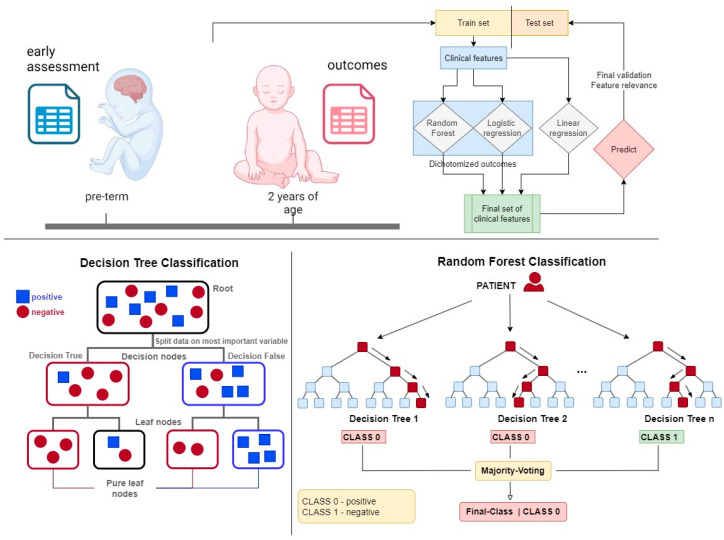
The upper schematic shows the concept of this work, which uses statistical and machine learning analysis to extract information on relevant prognostic features.

RF models [30] were trained using the scikit-learn library [31]. These models combine multiple weak nonlinear classifiers to enhance predictive capabilities and reduce prediction errors (see Figure 1). Nonlinear classifiers, such as RF, are particularly advantageous when dealing with heterogeneous data, where linear classifiers may underperform due to the complexity and variability inherent in medical data sets. RF models simplify the data preprocessing stage by reducing the need for extensive variable transformation. For instance, ordinal variables can be utilized directly without the need for binary encoding, which streamlines the model-building process and retains more information from the original data. This efficiency in handling diverse data types makes RF models particularly suitable for applications in pediatric disease prediction, as previously described [32,33]. To further enhance the interpretability and reliability of the models, we utilized permutation importance (PI) for model explanation and variable selection.

Permutation importance involves refitting pre-trained models in each iteration and shuffling the values of individual predictor variables. If a variable is crucial to the model, its performance will decline when the variable’s values are shuffled. This decline in performance is quantified and assigned as a weight towards the quality metrics. The greater a model’s reliance on a particular variable, the higher its associated weight, making it an effective method for identifying the most influential variables in predicting patient outcomes. We have successfully employed PI in our previous work to improve model transparency and ensure the robustness of our predictions [32,34]. For each outcome, all the predictive variables entered each model, i.e., RF, logistic, and linear regression. Then the variables were selected automatically using PI in the train set to remove insignificant variables. The data set was split per outcome into train and test sets (25%) and hence all reported models were trained on the train sets and evaluated on the respective test sets. A single hyperparameter was optimized using grid search for Random Forest, namely, the max depth (3,4,5) using the train set. During optimization variable selection was applied on the train set. Three metrics were used to validate model quality in the classification models: accuracy, Receiver Operating Characteristic Area Under the Curve (ROCAUC), and Matthews Correlation Coefficient (MCC). For the regression models, the R^2^ value was used.

### 2.5. Agreement with a Pediatric Opinion on Variable Importance

To understand the use of clinically relevant features and the feedback from ML, we compared the same set of predictive features used in this study (Table 1) with the rankings of 20 pediatricians. The pediatricians analyzed the given tables, ranking them based on their knowledge and clinical experience regarding their predictive capacity for the four BSID III outcomes (cognitive, language, motor, and socio-emotional). This will be compared with the feature importance of the ML models.

## 3. Results

### 3.1. Characteristics of the Overall Sample

A total of 64 preterm infants we included in the study. The mean GA at birth was 28.9 weeks (range 24.3–34.0, SD ± 2.36); 30 (47%) were girls, and 34 (53%) were boys. The mean birth weight was 1253 g (range 593–2810, SD ± 398.91). Perinatal and clinical characteristics of the study cohort are shown in Table 2. The number of infants with normal Bayley-III outcome (according to each scale) at the age of 2 years CA is shown in Table 3.

The results are split into subsections based on algorithms and type of outcome since the purpose of our work was to analyze the predictability of the outcomes. In every predictive model, all 30 variables were included in the analysis for each analyzed outcome (cognitive, language, motor, socio-emotional score). During model training, the variables were downselected using permutation importance as described previously.

### 3.2. Linear Regression Models for Numeric Outcomes

**Figure 2:**
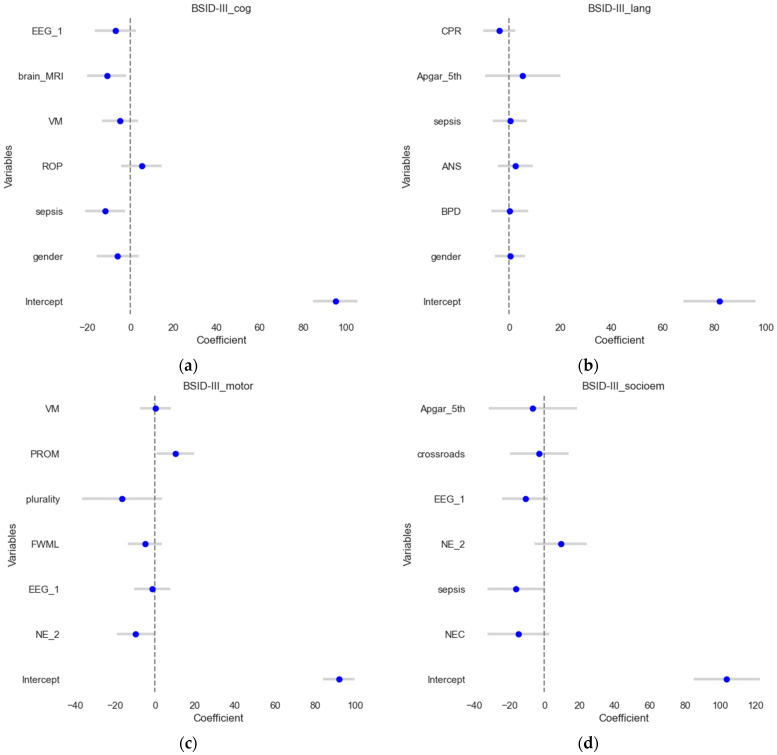
Forest plots of the linear regression results. The estimates or coefficients per model are shown on the *x*-axis, while the variables are presented on the *y*-axis in each subplot. The blue dot is the estimate, and the grey line is the confidence interval. The presented variables are those that have passed the variable selection procedure on the train set for (**a**) the BSID III cognitive outcome; (**b**) the BSID III language outcome; (**c**) the BSID III motoric scores and (**d**). BSID III socio-emotional assessment.

The numeric Bayley-III (BSID) scores were set as outcomes in these four multivariate linear regression models while utilizing feature selection by permutation importance. The four model’s results are depicted in Figure 2. The test set (a more rigorous procedure) is used to visualize the estimates and their confidence intervals. In all models, outliers were automatically removed by pre-set criteria. The model for BSID III cognitive score (Figure 2a) explained a substantial proportion of the variance in cognitive scores (R^2^ = 0.49, adjusted R^2^ = 0.31). For BSID III language scores (Figure 2b), the R^2^ value of 0.23 indicates that the model can explain 23% of the variance. However, the adjusted R-squared of zero suggests that the model does not fit the data well. Linear regression for BSID III motor scores (Figure 2c) explains 56% of the variance in motor scores (R^2^ = 0.56, adjusted R^2^ = 0.41). In contrast, the results for BSID III socio-emotional assessment (Figure 2d) display a strong fit (R^2^ = 0.63, adjusted R^2^ = 0.5) indicating that it explains 63% of the variance in socioemotional outcomes.

### 3.3. Logistic Regression for Dichotomized Outcomes

**Figure 3:**
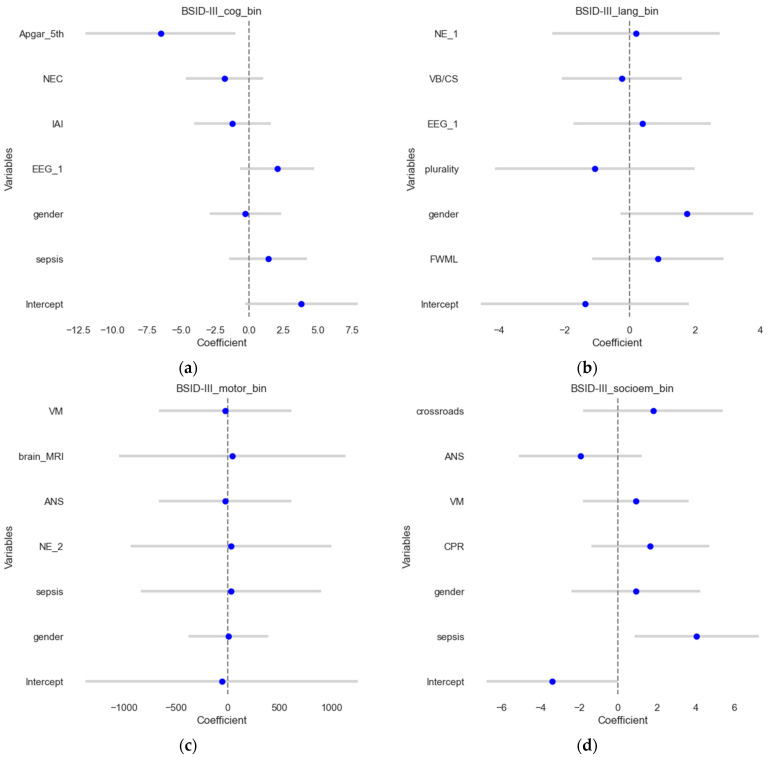
Forest plots of the logistic regression results. The estimates or coefficients per model are shown on the *x*-axis, while the variables are presented on the *y*-axis in each subplot. The blue dot is the estimate, and the grey line is the confidence interval. The results presented show logistic regression coefficients for (**a**) the BSID III cognitive outcome; (**b**) the BSID III language outcome; (**c**) the BSID III motoric scores and (**d**). BSID III socio-emotional assessment.

In this section, the binary outcomes were used as outcomes (contrary to the previous section) for the four scores, explained using multivariate logistic regression, and results were shown on the test sets. The results are shown in Figure 3, which visualizes the estimates and their confidence intervals. The model for the BSID III cognitive score (Figure 3a) demonstrated an accuracy of 0.58 and a ROCAUC of 0.56, which suggests that it predicts the outcome correctly in approximately 58% of the cases, and the ability to distinguish between the two classes is slightly better than a random guess. MCC was 0.12, suggesting poor predictive performance. For BSID III language outcomes, the model demonstrated an accuracy of 0.54, an ROCAUC of 0.52, and an MCC of 0.05. For BSID III motor scores, the model demonstrated an accuracy of 0.77, a ROCAUC of 0.72, and an MCC of 0.47, showing good agreement. Meanwhile, for the BSID III socio-emotional assessment, the models demonstrated an accuracy of 0.77, ROCAUC of 0.62, and MCC of 0.43, suggesting a reasonable predictive performance.

### 3.4. Random Forest Classification Models

In this subsection, we evaluated whether more complex ML models can reveal additional patterns and highlight additional features compared to previous simpler models. Each dichotomized outcome was used in RF models and underwent feature selection using permutation importance. The predictive quality is presented by their accuracy, ROCAUC, and MCC values in Table 4, according to each assessment category. The MCC shows that cognitive and motor scores can be predicted with reasonable quality.

During model training, features were selected based on permutation importance. Table 5 presents the final features per outcome.

The results show that sepsis and gestational age are predictors present in three models and duration of mechanical ventilation and hospitalization in two.

### 3.5. Statistical Tests

**Figure 4:**
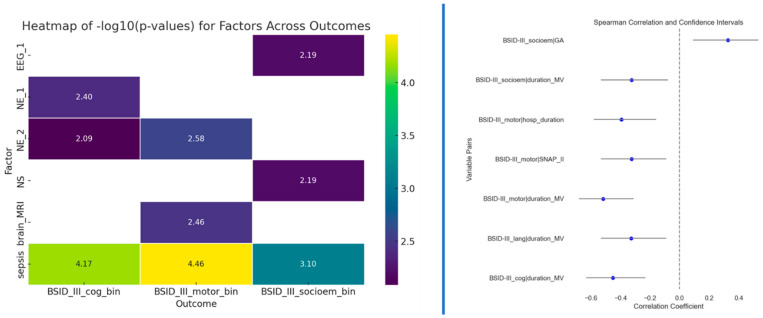
Left side: Results of the Fisher’s exact test between outcomes and predictors that passed the *p* < 0.01 threshold; Right side: Spearman correlation results for predictors that passed the *p* < 0.01 threshold.

Besides testing multivariate models, we also tested univariate associations for the four outcomes. Fisher’s exact test was used to determine potential associations between two categorical variables (to the outcomes), while Spearman’s correlation was used for continuous variables. The correlation results in Figure 4 (right side) indicate a significant association between the duration of mechanical ventilation (MV) and cognitive, motor, and socio-emotional outcomes. Notably, children who experienced shorter MV achieved better Bayley scores. Additionally, gestational age emerged as another statistically significant factor for socio-emotional outcomes. Longer gestation demonstrated significantly higher Bayley-III scores in the socio-emotional domain than those born earlier. Neurological examination at term and 12 months of CA and sepsis displayed significant associations with cognitive outcome. Sepsis, neurological examination at 12 months of CA, and abnormal MRI findings at TEA displayed significant associations with motoric outcome. Sepsis also had a significant association with socioemotional outcomes, as well as neonatal seizures and EEG at TEA. The presence of sepsis is likely to impact cognitive, motor, and socio-emotional outcomes in the studied population.

### 3.6. Clinician’s Opinion

Twenty pediatricians from Croatia were asked to evaluate all 30 predictive features used in the analyzed models to understand the importance of clinicians’ features compared to machines’ features. Table 6 shows the average ranks regarding the predictive capacity of each of the variables against the four outcomes.

## 4. Discussion

In this study, we demonstrated that, in comparison with linear classifiers, the nonlinear Random Forest (RF) model improved predictive accuracy and reduced errors in detecting variables significantly impacting Bayley outcomes at the age of 2 years.

Among all the analyzed predictive variables, linear classifiers identified sepsis, brain MRI abnormalities, Amiel-Tison neurological assessment at 12 months of CA, and Apgar score at 5th minute as having an impact on the prediction of the Bayley outcomes. Using the RF model, the most predictive variables for the Bayley outcomes were sepsis, GA, duration of MV and hospitalization, SNAPPE II, gender, Apgar score at 1st minute, focal lesion of white matter, Amiel-Tison neurological assessment at 12 months of CA, cardiopulmonary resuscitation, and EEG at term age.

Linear models may miss nonlinearities and interactions between predictors and outcomes. Additionally, due to the high heterogeneity of input variables, linear models can suffer from including irrelevant features and complex cancellation effects of these features [34,35,36]. In our analysis, the RF model outperformed linear classifiers by detecting a broader range of significant predictors for Bayley outcomes, highlighting its superiority in managing high-dimensional and heterogeneous data.

Using the RF approach, we can reach higher accuracies. The RF model achieved an accuracy of 77% for motor, cognitive, and socio-emotional Bayley domains and 65% for language, meaning the model correctly predicts the outcome in 77% and 65% of cases, respectively. In a study by Demirci et al., which included 1109 preterm infants, the highest balanced accuracy achieved at 19 months was similar: 72% for cognitive/language outcomes and 73% for motor outcomes [37]. Hence, this confirms the extent of our results given the cohort size and observed data. We advise the reader to keep the model accuracies in mind when assessing the relevance of the predictors in the latter parts of the discussion.

### 4.1. Predictors for Bayley Cognitive Outcome

Based on the linear regression model in our study, sepsis, and abnormal brain MRI at TEA were associated with decreased cognitive development. In contrast, the logistic regression model (dichotomized outcomes) showed that a low Apgar score at 5th minute was a negative predictor. The nonlinear, Random Forest (RF) approach identified sepsis as the strongest predictor for cognitive development, with contributions from GA, duration of invasive mechanical ventilation, and SNAPPE II score.

Infections in preterm infants, particularly recurrent ones, contribute to oligodendroglia injury and inhibition of maturation and myelination, including axonal damage and neuronal loss. These issues are linked to cognitive and attention impairments evident at school age and motor deficits with a higher risk of cerebral palsy development [38]. According to the literature, ventriculomegaly, focal white matter injury, and poor/no visible periventricular crossroads are predictors of neurodevelopmental impairment [8,9,16,38]. Our study’s linear regression analysis indicated that abnormal brain MRI was a predictor of cognitive impairment, which is consistent with previous findings. Interestingly, RF analysis did not identify abnormal total brain MRI or ventriculomegaly, crossroads visibility, or focal white matter injuries as significant predictors for cognitive outcomes. One possible reason for this discrepancy could be the small sample size or the infrequent occurrence of crossroads damage or focal white matter lesions on brain MRI. Another set of reasons can be due to differences in the algorithms, since RF is nonlinear and does not necessarily rely on associated variables but is rather splitting the data into subgroups.

Studies on the association between Apgar score and Bayley outcome have shown divergent results. Some studies indicate that a low Apgar score predicts cerebral palsy, ADHD, and gross motor delay [39,40,41]. In contrast, others suggest that a low Apgar score is not associated with long-term cognitive or motor impairment in preterm infants [42]. Hence, these associations could profit from further research. Similarly, the impact of the SNAP II score on cognitive outcomes has shown mixed results. A SNAP II score above 30 has been suggested as a possible negative predictor for cognitive development, lower academic achievements, behavioral problems, and gross motor function impairments [43]. Impaired cognitive outcome in preterm infants with higher SNAP/SNAPPE II scores may reflect their immaturity and vulnerability, respectively, emphasizing the importance of early health stability in long-term development [44].

### 4.2. Predictors for Bayley Motor Outcome

Amiel-Tison neurological assessment at 12 months of CA (NE_2) was the only statistically significant predictor of motor impairment according to our linear regression analysis. Based on our RF-classification approach, the presence of sepsis, focal lesions of white matter, abnormal Amiel-Tison neurological assessment at 12 months of CA, and cardiopulmonary resuscitation significantly predict motor impairment. By detecting more significant predictors, we notice that the nonlinear RF model enables a clearer selection of high-risk preterm infants.

White matter abnormalities on brain MRI at TEA, especially punctiform white matter lesions, are related to motor impairment [45]. We also confirmed that with our Random Forest method. Unlike literature data, in our study, not visible/slightly visible crossroads or ventriculomegaly were not associated with impaired motor development [8,9]. This result needs to be confirmed with analyses on the bigger cohort. Amiel-Tison neurological assessment at term age is a well-known predictor of short-term motor outcome [46,47]. According to our linear and nonlinear analysis, Amiel-Tison assessment at 12 months of CA was significantly associated with motor outcome at two years.

### 4.3. Predictors for Bayley Socio-Emotional Outcome

Among all predictive features in our study, based on linear regression and classification analyses, only sepsis negatively impacted socio-emotional development. The nonlinear RF method, in addition to sepsis, identified GA, duration of hospitalization, and EEG at TEA as predictors for socio-emotional outcome.

Negative and inverse effects of gestational age on neurodevelopment outcomes (cognitive, language, and socio-emotional) may be mediated by postnatal procedures and events that preterm infants with lower GA require [4,33,43,48].

EEG discontinuity, asymmetry, or epileptic discharges at TEA, especially in combination with brain structural abnormalities, are related to abnormal neurodevelopment [49], as shown in our machine learning results. Frontal EEG asymmetry could be a predictor for behavioral problems in stress regulation in infants born at extremely low birth weight [50]. The grade of EEG abnormalities correlates with the incidence of behavioral difficulties in preterm infants [51]. Our study did not analyze the correlation between the grade of EEG abnormalities and the incidence of neurodevelopmental delay, so this will be our future step.

Longer hospitalization, in addition to cognitive and motor impairment, can result in language and behavioral difficulties at the corrected age of two. However, the effect on long-term global development outcomes remains of questionable significance, and a longer follow-up period is required [52].

### 4.4. Predictors for Bayley Language Outcome

Using linear or logistic regression model analysis, none of the predictors showed a statistically significant association with the language outcome in our study. However, based on the ML model, the key predictor of poor language outcome was a longer hospitalization, followed by a low Apgar score at 1st minute, GA, and a longer duration of invasive mechanical ventilation (IMV).

The negative correlation between the duration of IMV and cognitive, motor, or language development reminds us that the minimal duration of IMV should be an important focus in preterm infant treatment [53].

Some previous longitudinal studies have shown a negative association between language development and socioeconomic factors such as maternal education, home quality, social interaction, and family income [54,55]. We did not analyze socioeconomic factors in our study. Future studies should incorporate these variables to provide a more holistic understanding of developmental risks and outcomes, potentially leading to more tailored and effective interventions.

In a paper by Tseng et al., neonatal sepsis was also identified as a risk factor for severe language delay. Still, we did not find a statistically significant predictive impact in our work [56].

### 4.5. Discrepancy with Pediatricians’ Opinions

Our study highlights significant discrepancies between the pediatricians’ opinions and the findings from our machine learning (ML) models regarding predictors of cognitive, motor, socio-emotional, and language outcomes in preterm infants. Among the same set of predictive variables included in our study, pediatricians identified cardiopulmonary resuscitation as a key predictor for cognitive, motor, and socio-emotional outcomes and GA for language outcomes. Conversely, our ML analysis identified sepsis as the primary predictor for cognitive and motor outcomes, while GA and duration of hospitalization were significant predictors for socio-emotional outcomes. For language outcome, the ML model emphasized the duration of hospitalization, low Apgar score at 1st minute, and GA as a key predictor. This partially coincides with pediatrics’ opinions regarding GA as a key predictor. To our knowledge, this is the first time a comparison between clinicians’ opinions and the machine learning approach has been made in this setting.

These discrepancies between ML models and pediatricians underline the complexity of predicting developmental outcomes in preterm infants and the evolving nature of research in this field. Existing literature provides mixed findings, often influenced by study design, sample size, and analytical methods. For instance, while some studies have identified sepsis as a significant predictor of poor neurodevelopmental outcomes, others highlight the role of socioeconomic factors, which were not analyzed in our study but are well-documented in the literature.

Our ML approach aligns with some aspects of previous research by identifying sepsis, GA, and duration of hospitalization or invasive mechanical ventilation as critical predictors. However, the ML model’s failure to highlight cardiopulmonary resuscitation as a key predictor for cognitive, motor, or socio-emotional outcomes, despite its significance in clinical practice, suggests that ML models may sometimes miss clinically relevant factors due to limitations such as small sample sizes or the nuances of clinical judgment that are not easily quantifiable. Machine learning offers a data-driven approach that can uncover complex, nonlinear relationships between predictors and outcomes, often beyond the capacity of traditional statistical methods. However, the insights from these models should complement, not replace, clinical expertise.

Our study underscores the need for interdisciplinary collaboration in healthcare. By combining the strengths of machine learning with the nuanced understanding of experienced clinicians, we can develop more robust predictive models. This approach enhances clinical decision-making and ensures that predictive models remain clinically relevant and grounded in real-world practice. Moreover, the divergence in findings between pediatricians’ opinions and ML results points to the potential of machine learning as a tool to challenge and refine clinical assumptions. Pediatricians should consider research data when making predictions to avoid potential biases and ensure that the most current evidence supports their decisions.

### 4.6. Strengths and Limitations

The strength of our study is that it predicted preterm infants’ outcomes in all four Bayley domains using a varied set of variables. Many known research findings predict outcomes in fewer domains [37,55,57,58,59]. Predicting outcomes in different domains enables robust backup in clinical decisions to choose appropriate therapeutic approaches. On the other hand, early and accurate identification of risk preterm infants will make early interventions possible in neonatal intensive care units.

While our study underscores the strengths of machine learning, it also highlights certain limitations. The absence of socioeconomic data, potential small cohort size, and the need for longitudinal follow-up are areas that warrant further investigation.

Future research should aim to incorporate a broader range of variables, including socioeconomic factors, to provide a more comprehensive understanding of predictors of developmental outcomes. Longitudinal studies with larger sample sizes and longer follow up, at least to school age, can help validate the findings from machine learning models and explore the long-term effects of identified predictors.

Additionally, continuous training and education for healthcare professionals on the capabilities and limitations of machine learning can facilitate its integration into clinical practice, ultimately leading to improved care for preterm infants.

## 5. Conclusions

Our study highlights the transformative potential of machine learning in neonatal care, offering new avenues for research and clinical practice.

Among all analyzed predictors in our study, linear classifiers identified sepsis, total brain MRI findings, and Apgar score at 5th minute as predictive for cognitive, Amiel-Tison neurological assessment at 12 months of CA for motor, and again sepsis for socio-emotional outcome. None of the analyzed features were predictive for language outcome. Machine learning analysis showed that sepsis was the key predictor for cognitive and motor outcome. For language, equally predictive were gestational age, duration of hospitalization, and Apgar score at 1st minute, while for socio-emotional outcome, gestational age, sepsis, and duration of hospitalization were predictive.

Among the same set of predictors included in our study analyses, pediatricians identified cardiopulmonary resuscitation as a key predictor for cognitive, motor, and socio-emotional, and gestational age as predictive for language outcomes. These discrepancies between ML models and pediatricians underline the complexity of predicting developmental outcomes in preterm infants and the evolving nature of research in this field.

Machine learning methods should be viewed as a complement to, rather than a replacement for, clinical expertise. Pediatricians can ensure that their predictions and interventions are both scientifically sound and practically effective by aligning their clinical practices with the latest research findings. By bridging the gap between empirical data and clinical experience, we can develop more effective and personalized approaches to support the development of preterm infants, ensuring they achieve their fullest potential.

## Figures and Tables

**Table 1:** Predictive features used for statistical and machine learning analysis.

Features Group	Predictive Variables
Pregnancy and birth data	method of conception, birth (vaginal/cesarean section), plurality, ANS, IAI, gender, GA, BW, IUGR
Clinical characteristics	Apgar score at 1st and 5th minute, CPR, duration of invasive MV, sepsis, SNAP II, SNAPPE II, BPD, NEC, ROP, PDA, NS, duration of hospitalization (in days)
MRI at TEA	ventriculomegaly, visibility of frontal and parietal crossroads, focal white matter injuries, MRI finding at TEA
cEEG	cEEG at TEA (EEG1) and at 12 months of CA (EEG2)
Neurological assessment	Amiel-Tison examination at TEA (NE1) and 12 months of CA (NE2)

Abbreviations: antenatal steroid administration (ANS), bronchopulmonary dysplasia (BPD), birth weight (BW), corrected age (CA), cardiopulmonary resuscitation (CPR), electroencephalogram (EEG), gestational age (GA), intraamniotic infection (IAI), intrauterine growth restriction (IUGR), mechanical ventilation (MV), necrotizing enterocolitis (NEC), neonatal epileptic seizures (NS), persistent ductus arteriosus (PDA), premature retinopathy (ROP), term equivalent age (TEA), Score for Neonatal Acute Physiology (SNAP II), SNAP Perinatal Extension (SNAPPE II).

**Table 2:** Perinatal and clinical characteristics of the study cohort (N = 64).

Characteristics	N	%
Plurality	15	23
IVF	9	14
Vaginal birth	22	34
Cesarean section	42	66
BPD	20	31
NEC	17	27
ROP	47	73
Apgar 1st min < 7	29	45
Apgar 5th min < 7	20	31
ANS	39	61
IAI	11	17
IUGR	4	9
CPR	20	31
Sepsis	25	39
PDA	9	14
NS	12	19

Abbreviation: antenatal steroid administration (ANS), bronchopulmonary dysplasia (BPD), cardiopulmonary resuscitation (CPR), intraamniotic infection (IAI), intrauterine growth restriction (IUGR), IVF (in vitro fertilization), necrotizing enterocolitis (NEC), neonatal epileptic seizures (NS), persistent ductus arteriosus (PDA), premature retinopathy (ROP).

**Table 3:** Bayley III scale outcomes of the study cohort (N = 64).

Bayley III ≥ 85 Score	N	%
Language scale	40	63
Motor scale	41	64
Socio-emotional scale	52	81
Cognitive scale	38	59

**Table 4:** Model Performance Metrics in BSID III Assessments.

Assessment Category	Accuracy	ROCAUC	MCC
Cognitive Score	0.77	0.76	0.53
Language Assessment	0.65	0.62	0.26
Motor Scores	0.77	0.80	0.57
Socio-emotional Assessment	0.77	0.70	0.36

**Table 5:** Feature Importance in BSID III Assessments.

Feature	Cognitive Outcome (%)	Language Outcome (%)	Motor Outcome (%)	Socio-Emotional Outcome (%)
Sepsis	30	0	60	30
GA	20	30	-	30
Duration MV	20	20	-	-
SNAPPE II	20	-	-	-
Gender	10	-	-	-
Duration of hospitalization	-	30	-	30
Apgar 1st minute	-	30	-	-
FWML	-	-	20	-
NE 2	-	-	10	-
CPR	-	-	10	-
EEG 1	-	-	-	10

Abbreviation: cardiopulmonary resuscitation (CPR), electroencephalogram at term-equivalent age (EEG 1), focal white matter lesion (FWML), gestational age (GA), mechanical ventilation (MV), neurological examination at 12 months of corrected age (NE2), Score for Neonatal Acute Physiology Perinatal Extension (SNAPPE II).

**Table 6:** Average ranks of each feature regarding predictive capacity for four Bayley-III domains (cognitive, motor, socio-emotional, language).

Bayley-III Cognitive		Bayley-III Motor		Bayley-III Socio-Emotional		Bayley-III Language	
CPR	1	CPR	1	CPR	1	GA	1
GA	2	GA	2	Apgar 5th min.	2	CPR	2
Apgar 5th min.	3	FWML	3	GA	3	Apgar 1st min.	3
sepsis	4	Apgar 5th min.	4	Apgar 1st min.	4	Apgar 5th min.	4
Apgar 1st min.	5	Apgar 1st min.	5	BW	5	FWML	5
NS	6	brain MRI	6	sepsis	6	duration MV	6
crossroads	7	crossroads	7	NS	7	SNAPPE II	7
SNAPPE II	8	VM	8	SNAP II	8	BW	8
FWML	9	duration MV	9	duration MV	9	brain MRI	9
BW	10	sepsis	10	SNAPPE II	10	NE 1	10
duration MV	11	NS	11	FWML	11	sepsis	11
SNAP II	12	SNAPPE II	12	NE 1	12	SNAP II	12
Brain MRI	13	IUGR	13	EEG 1	13	NS	13
VM	14	NE 1	14	crossroads	14	EEG 1	14
BPD	15	BW	15	DH	15	VM	15
NE 1	16	EEG 1	16	VM	16	IUGR	16
NE 2	17	IAI	17	brain MRI	17	NE 2	17
IUGR	18	NE 2	18	NE 2	18	EEG 2	18
NEC	19	SNAP II	19	IUGR	19	crossroads	19
EEG 1	20	BPD	20	EEG 2	20	BPD	20
EEG 2	21	DH	21	IAI	21	IAI	21
DH	22	EEG 2	22	BPD	22	DH	22
IAI	23	NEC	23	NEC	23	plurality	23
PDA	24	ANS	24	ANS	24	NEC	24
ANS	25	ROP	25	PDA	25	gender	25
ROP	26	PDA	26	plurality	26	ANS	26
plurality	27	VB/CS	27	gender	27	PDA	27
gender	28	gender	28	ROP	28	ROP	28
VB/CS	29	plurality	29	VB/CS	29	IVF	29
IVF	30	IVF	30	IVF	30	VB/CS	30

Abbreviations: antenatal steroid administration (ANS), bronchopulmonary dysplasia (BPD), birth weight (BW), cesarean section (CS), cardiopulmonary resuscitation (CPR), duration of hospitalization (DH), electroencephalogram at term equivalent age (EEG1), electroencephalogram at 12 months of corrected age (EEG2), focal white matter lesion, (FWML), gestational age (GA), intraamniotic infection (IAI), intrauterine growth restriction (IUGR), in vitro fertilization (IVF), mechanical ventilation (MV), magnetic resonance imaging (MRI), neurological examination at term equivalent age (NE1), neurological examination at 12 months of corrected age (NE2), necrotizing enterocolitis (NEC), neonatal epileptic seizures (NS), persistent ductus arteriosus (PDA), premature retinopathy (ROP), Score for Neonatal Acute Physiology (SNAP II), SNAP Perinatal Extension (SNAPPE II), vaginal birth (VB), ventriculomegaly (VM).

## Data Availability

For reasons of data privacy and protection, the data are not available.

## References

[1] Cao G., Liu J., Liu M. (2022). Global, Regional, and National Incidence and Mortality of Neonatal Preterm Birth, 1990–2019. JAMA Pediatr..

[2] Hee Chung E., Chou J., Brown K.A. (2020). Neurodevelopmental Outcomes of Preterm Infants: A Recent Literature Review. Transl. Pediatr..

[3] Ream M.A., Lehwald L. (2018). Neurologic Consequences of Preterm Birth. Curr. Neurol. Neurosci. Rep..

[4] WHO Preterm Birth. https://www.who.int/news-room/fact-sheets/detail/preterm-birth.

[5] Do C.H.T., Kruse A.Y., Wills B., Sabanathan S., Clapham H., Pedersen F.K., Pham T.N., Vu P.M., Børresen M.L. (2020). Neurodevelopment at 2 Years Corrected Age among Vietnamese Preterm Infants. Arch. Dis. Child..

[6] Lloyd R.O., O’Toole J.M., Livingstone V., Filan P.M., Boylan G.B. (2021). Can EEG Accurately Predict 2-Year Neurodevelopmental Outcome for Preterm Infants?. Arch. Dis. Child. Fetal Neonatal Ed..

[7] Agut T., Alarcon A., Cabañas F., Bartocci M., Martinez-Biarge M., Horsch S. (2020). Preterm White Matter Injury: Ultrasound Diagnosis and Classification. Pediatr. Res..

[8] Judas M., Rados M., Jovanov-Milosevic N., Hrabac P., Stern-Padovan R., Kostovic I. (2005). Structural, Immunocytochemical, and Mr Imaging Properties of Periventricular Crossroads of Growing Cortical Pathways in Preterm Infants. AJNR Am. J. Neuroradiol..

[9] Bunoza B., Barišić N., Grđan Stevanović P., Bogdanić A., Benjak V., Grizelj R., Turudić D., Milošević D., Radoš M. (2021). The Visibility of the Periventricular Crossroads of Pathways in Preterm Infants as a Predictor of Neurological Outcome and Occurrence of Neonatal Epileptic Seizures. Croat. Med. J..

[10] Sheng M., Guo T., Mabbott C., Chau V., Synnes A., de Vries L.S., Grunau R.E., Miller S.P. (2022). Ventricular Volume in Infants Born Very Preterm: Relationship with Brain Maturation and Neurodevelopment at Age 4.5 Years. J. Pediatr..

[11] Gosselin J., Gahagan S., Amiel-Tison C. (2005). The Amiel-Tison Neurological Assessment at Term: Conceptual and Methodological Continuity in the Course of Follow-Up. Ment. Retard. Dev. Disabil. Res. Rev..

[12] Paro-Panjan D., Neubauer D., Kodrie J., Bratanic B. (2005). Amiel-Tison Neurological Assessment at Term Age: Clinical Application, Correlation with Other Methods, and Outcome at 12 to 15 Months. Dev. Med. Child Neurol..

[13] Jobe A.H., Bancalari E. (2001). Bronchopulmonary Dysplasia. Am. J. Respir. Crit. Care Med..

[14] Pressler R.M., Cilio M.R., Mizrahi E.M., Moshé S.L., Nunes M.L., Plouin P., Vanhatalo S., Yozawitz E., de Vries L.S., Puthenveettil Vinayan K. (2021). The ILAE Classification of Seizures and the Epilepsies: Modification for Seizures in the Neonate. Position Paper by the ILAE Task Force on Neonatal Seizures. Epilepsia.

[15] Yoo Y., Park J.-E., Park M., Lee J. (2021). Implementation of the Feed and Swaddle Technique as a Non-Pharmacological Strategy to Conduct Brain Magnetic Resonance Imaging in Very Low Birth Weight Infants. Neonatal Med..

[16] Kostović I., Kostović-Srzentić M., Benjak V., Jovanov-Milošević N., Radoš M. (2014). Developmental Dynamics of Radial Vulnerability in the Cerebral Compartments in Preterm Infants and Neonates. Front. Neurol..

[17] Milos R.-I., Jovanov-Milošević N., Mitter C., Bobić-Rasonja M., Pogledic I., Gruber G.M., Kasprian G., Brugger P.C., Weber M., Judaš M. (2020). Developmental Dynamics of the Periventricular Parietal Crossroads of Growing Cortical Pathways in the Fetal Brain—In Vivo Fetal MRI with Histological Correlation. Neuroimage.

[18] Kidokoro H., Neil J.J., Inder T.E. (2013). New MR Imaging Assessment Tool to Define Brain Abnormalities in Very Preterm Infants at Term. AJNR Am. J. Neuroradiol..

[19] Miller S.P., Cozzio C.C., Goldstein R.B., Ferriero D.M., Partridge J.C., Vigneron D.B., Barkovich A.J. (2003). Comparing the Diagnosis of White Matter Injury in Premature Newborns with Serial MR Imaging and Transfontanel Ultrasonography Findings. AJNR Am. J. Neuroradiol..

[20] Bulbul L., Elitok G.K., Ayyıldız E., Kabakcı D., Uslu S., Köse G., Tiryaki Demir S., Bulbul A. (2020). Neuromotor Development Evaluation of Preterm Babies Less than 34 Weeks of Gestation with Bayley III at 18–24 Months. BioMed Res. Int..

[21] Çelik P., Ayranci Sucakli I., Yakut H.I. (2020). Which Bayley-III Cut-off Values Should Be Used in Different Developmental Levels?. Turk. J. Med. Sci..

[22] Fernandes L.V., Goulart A.L., dos Santos A.M.N., Barros M.C.M., Guerra C.C., Kopelman B.I. (2012). Neurodevelopmental Assessment of Very Low Birth Weight Preterm Infants at Corrected Age of 18–24 Months by Bayley III Scales. J. Pediatr..

[23] Kenyhercz F., Nagy B.E. (2022). A New Perspective: Establishing Developmental Profiles of Premature Infants Based on Bayley-III Scores at Age 2. Appl. Neuropsychol. Child.

[24] Månsson J., Källén K., Eklöf E., Serenius F., Ådén U., Stjernqvist K. (2021). The Ability of Bayley-III Scores to Predict Later Intelligence in Children Born Extremely Preterm. Acta Paediatr..

[25] Virtanen P., Gommers R., Oliphant T.E., Haberland M., Reddy T., Cournapeau D., Burovski E., Peterson P., Weckesser W., Bright J. (2020). SciPy 1.0: Fundamental Algorithms for Scientific Computing in Python. Nat. Methods.

[26] Perktold J., Seabold S. Econometric and Statistical Modeling with Python Python. Proceedings of the 9th Python in Science Conference.

[27] Vallat R. (2018). Pingouin: Statistics in Python. J. Open Source Softw..

[28] Hunter J.D. (2007). Matplotlib: A 2D Graphics Environment. Comput. Sci. Eng..

[29] Waskom M.L. (2021). Seaborn: Statistical Data Visualization. J. Open Source Softw..

[30] Breiman L. (2001). Random Forests. Mach. Learn..

[31] Pedregosa F., Varoquaux G., Gramfort A., Michel V., Thirion B., Grisel O., Blondel M., Prettenhofer P., Weiss R., Dubourg V. (2011). Scikit-Learn: Machine Learning in Python. J. Mach. Learn. Res..

[32] Lovrić M., Banić I., Lacić E., Pavlović K., Kern R., Turkalj M. (2021). Predicting Treatment Outcomes Using Explainable Machine Learning in Children with Asthma. Children.

[33] van Boven M.R., Henke C.E., Leemhuis A.G., Hoogendoorn M., van Kaam A.H., Königs M., Oosterlaan J. (2022). Machine Learning Prediction Models for Neurodevelopmental Outcome after Preterm Birth: A Scoping Review and New Machine Learning Evaluation Framework. Pediatrics.

[34] Lundberg S.M., Erion G., Chen H., DeGrave A., Prutkin J.M., Nair B., Katz R., Himmelfarb J., Bansal N., Lee S.-I. (2020). From Local Explanations to Global Understanding with Explainable AI for Trees. Nat. Mach. Intell..

[35] Lovrić M., Horner D., Chen L., Brustad N., Malby Schoos A.-M., Lasky-Su J., Chawes B., Rasmussen M.A. (2024). Vertical Metabolome Transfer from Mother to Child: An Explainable Machine Learning Method for Detecting Metabolomic Heritability. Metabolites.

[36] Lovric M., Pavlović K., Žuvela P., Spataru A., Lucic B., Kern R., Wong M. (2021). Machine Learning in Prediction of Intrinsic Aqueous Solubility of Drug-like Compounds: Generalization, Complexity, or Predictive Ability?. J. Chemom..

[37] Demirci G.M., Kittler P.M., Phan H.T.T., Gordon A.D., Flory M.J., Parab S.M., Tsai C.-L. (2024). Predicting Mental and Psychomotor Delay in Very Pre-Term Infants Using Machine Learning. Pediatr. Res..

[38] Sewell E., Roberts J., Mukhopadhyay S. (2021). Association of Infection in Neonates and Long-Term Neurodevelopmental Outcome. Clin. Perinatol..

[39] Hirvonen M., Ojala R., Korhonen P., Haataja P., Eriksson K., Gissler M., Luukkaala T., Tammela O. (2014). Cerebral Palsy among Children Born Moderately and Late Preterm. Pediatrics.

[40] Lindström K., Lindblad F., Hjern A. (2011). Preterm Birth and Attention-Deficit/Hyperactivity Disorder in Schoolchildren. Pediatrics.

[41] Hassen T.A., Chojenta C., Egan N., Loxton D. (2021). The Association between the Five-Minute Apgar Score and Neurodevelopmental Outcomes among Children Aged 8–66 Months in Australia. Int. J. Environ. Res. Public Health.

[42] Ehrhardt H., Aubert A.M., Ådén U., Draper E.S., Gudmundsdottir A., Varendi H., Weber T., Zemlin M., Maier R.F., Zeitlin J. (2023). Apgar Score and Neurodevelopmental Outcomes at Age 5 Years in Infants Born Extremely Preterm. JAMA Netw. Open.

[43] Logan J.W., Dammann O., Allred E.N., Dammann C., Beam K., Joseph R.M., O’Shea T.M., Leviton A., Kuban K.C.K. (2017). Early Postnatal Illness Severity Scores Predict Neurodevelopmental Impairments at 10 Years of Age in Children Born Extremely Preterm. J. Perinatol..

[44] Dammann O., Naples M., Bednarek F., Shah B., Kuban K.C.K., O’Shea T.M., Paneth N., Allred E.N., Leviton A., ELGAN Study Investigators (2010). SNAP-II and SNAPPE-II and the Risk of Structural and Functional Brain Disorders in Extremely Low Gestational Age Newborns: The ELGAN Study. Neonatology.

[45] Anderson P.J., Treyvaud K., Neil J.J., Cheong J.L.Y., Hunt R.W., Thompson D.K., Lee K.J., Doyle L.W., Inder T.E. (2017). Associations of Newborn Brain Magnetic Resonance Imaging with Long-Term Neurodevelopmental Impairments in Very Preterm Children. J. Pediatr..

[46] Herbón F., Garibotti G., Moguilevsky J. (2015). Predicción temprana del resultado neurológico a los 12 meses en neonatos de riesgo en Bariloche. An. Pediatr..

[47] Leroux B.G., N’Guyen The Tich S., Branger B., Gascoin G., Rouger V., Berlie I., Montcho Y., Ancel P.-Y., Rozé J.-C., Flamant C. (2013). Neurological Assessment of Preterm Infants for Predicting Neuromotor Status at 2 Years: Results from the LIFT Cohort. BMJ Open.

[48] Juul S.E., Wood T.R., German K., Law J.B., Kolnik S.E., Puia-Dumitrescu M., Mietzsch U., Gogcu S., Comstock B.A., Li S. (2023). Predicting 2-Year Neurodevelopmental Outcomes in Extremely Preterm Infants Using Graphical Network and Machine Learning Approaches. eClinicalMedicine.

[49] Yerushalmy-Feler A., Marom R., Peylan T., Korn A., Haham A., Mandel D., Yarkoni I., Bassan H. (2014). Electroencephalographic Characteristics in Preterm Infants Born with Intrauterine Growth Restriction. J. Pediatr..

[50] Schmidt L.A., Miskovic V., Boyle M., Saigal S. (2010). Frontal Electroencephalogram Asymmetry, Salivary Cortisol, and Internalizing Behavior Problems in Young Adults Who Were Born at Extremely Low Birth Weight. Child Dev..

[51] Hayashi-Kurahashi N., Kidokoro H., Kubota T., Maruyama K., Kato Y., Kato T., Natsume J., Hayakawa F., Watanabe K., Okumura A. (2012). EEG for Predicting Early Neurodevelopment in Preterm Infants: An Observational Cohort Study. Pediatrics.

[52] Badr L.K., Bookheimer S., Purdy I., Deeb M. (2009). Predictors of Neurodevelopmental Outcome for Preterm Infants with Brain Injury: MRI, Medical and Environmental Factors. Early Hum. Dev..

[53] Vliegenthart R.J.S., van Kaam A.H., Aarnoudse-Moens C.S.H., van Wassenaer A.G., Onland W. (2019). Duration of Mechanical Ventilation and Neurodevelopment in Preterm Infants. Arch. Dis. Child. Fetal Neonatal Ed..

[54] Pérez-Pereira M., Fernández P., Resches M., Gómez-Taibo M.L. (2013). Determinants of early language and communication in preterm and full term infants: A comparative study. Enfance.

[55] Valavani E., Blesa M., Galdi P., Sullivan G., Dean B., Cruickshank H., Sitko-Rudnicka M., Bastin M.E., Chin R.F.M., MacIntyre D.J. (2022). Language Function Following Preterm Birth: Prediction Using Machine Learning. Pediatr. Res..

[56] Tseng W.-L., Chen C.-H., Chang J.-H., Peng C.-C., Jim W.-T., Lin C.-Y., Hsu C.-H., Liu T.-Y., Chang H.-Y., on behalf of The Taiwan Premature Infant Follow-Up Network (2023). Risk Factors of Language Delay at Two Years of Corrected Age among Very-Low-Birth-Weight Preterm Infants: A Population-Based Study. Children.

[57] Ambalavanan N., Baibergenova A., Carlo W.A., Saigal S., Schmidt B., Thorpe K.E., Trial of Indomethacin Prophylaxis in Preterms (TIPP) Investigators (2006). Early Prediction of Poor Outcome in Extremely Low Birth Weight Infants by Classification Tree Analysis. J. Pediatr..

[58] Saha S., Pagnozzi A., Bourgeat P., George J.M., Bradford D., Colditz P.B., Boyd R.N., Rose S.E., Fripp J., Pannek K. (2020). Predicting Motor Outcome in Preterm Infants from Very Early Brain Diffusion MRI Using a Deep Learning Convolutional Neural Network (CNN) Model. Neuroimage.

[59] Vassar R., Schadl K., Cahill-Rowley K., Yeom K., Stevenson D., Rose J. (2020). Neonatal Brain Microstructure and Machine-Learning-Based Prediction of Early Language Development in Children Born Very Preterm. Pediatr. Neurol..

